# Selenium deficiency-induced cardiomyopathy: a case report

**DOI:** 10.1093/ehjcr/ytag184

**Published:** 2026-03-10

**Authors:** Tomonari Moriizumi, Takahiko Nishiyama, Shun Kohsaka, Masaki Ieda

**Affiliations:** Department of Cardiology, Keio University School of Medicine, 35 Shinanomachi, Shinjuku-ku, Tokyo 160-8582, Japan; Department of Cardiology, Keio University School of Medicine, 35 Shinanomachi, Shinjuku-ku, Tokyo 160-8582, Japan; Department of Cardiology, Keio University School of Medicine, 35 Shinanomachi, Shinjuku-ku, Tokyo 160-8582, Japan; Department of Cardiology, Keio University School of Medicine, 35 Shinanomachi, Shinjuku-ku, Tokyo 160-8582, Japan

**Keywords:** Case report, Crohn’s disease, Non-ischaemic cardiomyopathy, Haemodialysis, Myocardial biopsy, Selenium

## Abstract

**Background:**

Selenium is an essential trace element involved in redox homeostasis, thyroid function, and immune regulation. Deficiency is uncommon in developed countries but occurs in patients with malabsorption, long-term parenteral nutrition, or chronic kidney disease on dialysis. Selenium deficiency is a rare but treatable cause of non-ischaemic cardiomyopathy and arrhythmias, which can be fatal if untreated. Due to its rarity, it is often unrecognized, its pathophysiology remains unclear, and the threshold for irreversible myocardial damage is not well defined.

**Case summary:**

We present the case of a 63-year-old man with Crohn’s disease and progressive renal failure admitted for haemodialysis initiation. Over 3 months, a rapid decline in cardiac function was observed, and an electron microscopic study of the myocardial biopsy specimen revealed marked myocardial cell degeneration. Subsequent detailed physical examination suggested leukonychia, and laboratory tests confirmed low serum selenium levels. Following selenium supplementation, his cardiac function and symptoms of heart failure improved.

**Discussion:**

This case highlights the importance of considering selenium deficiency in the differential diagnosis of non-ischaemic cardiomyopathy, particularly in patients with risk factors such as intestinal malabsorption and dialysis. Early recognition and targeted supplementation can result in complete recovery of cardiac function, even in cases with histological evidence of structural myocardial degeneration.

Learning points

- Selenium deficiency should be considered in patients with unexplained non-ischaemic cardiomyopathy.
- Risk factors include Crohn’s disease, extensive intestinal resection, end-stage renal disease on dialysis, chronic inflammation, and long-term parenteral nutrition.
- Even in cases with marked myocardial injury, selenium deficiency-induced cardiomyopathy can be reversible with appropriate supplementation.

## Introduction

Selenium is an essential trace element involved in antioxidant defence, immune regulation, and thyroid function. Deficiency is rare in developed countries, but occurs in patients with Crohn’s disease, extensive intestinal resection, long-term parenteral nutrition, or end-stage renal disease requiring haemodialysis.^1^

Selenium deficiency-induced cardiomyopathy is characterized by cardiac dysfunction that may resemble dilated cardiomyopathy and can be fatal if not promptly recognized and treated.^2^ Although early diagnosis is challenging owing to non-specific symptoms, it is critical because appropriate supplementation may reverse cardiac dysfunction.

The pathophysiology involves impaired glutathione peroxidase activity, resulting in increased oxidative stress, mitochondrial dysfunction, and cardiomyocyte injury.^3^ Most previous reports have mainly described cases in patients receiving total parenteral nutrition or those with severe malabsorption; however, those combining multiple risk factors are rare.^4^

Herein, we report a rare case of cardiomyopathy from selenium deficiency in a patient with long-standing Crohn’s disease, multiple ileal resections, and end-stage renal disease. Despite severe myocardial structural abnormalities, the cardiac function significantly improved after selenium supplementation. This case highlights the importance of considering trace element deficiencies in the differential diagnosis of acute cardiac dysfunction, particularly in patients with multiple risk factors.

## Summary figure

**Figure:**
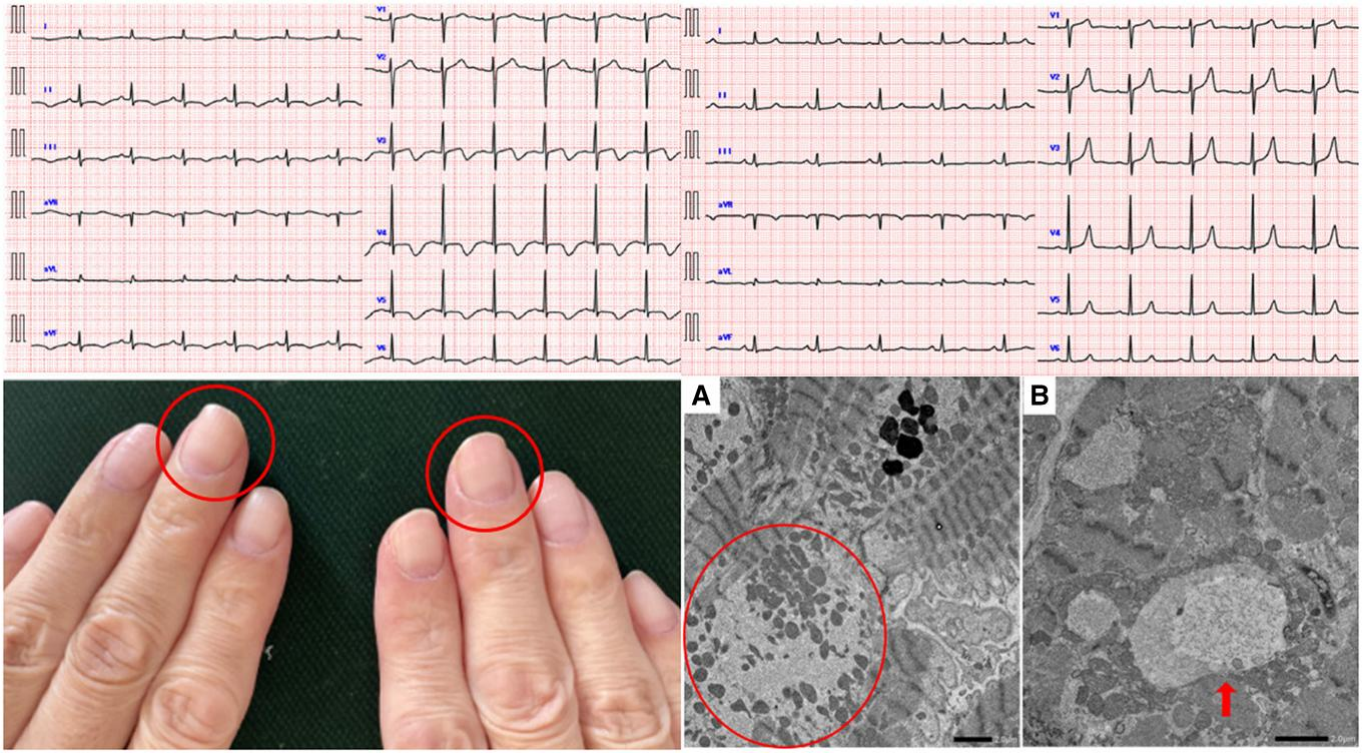
Multipanel image of key clinical findings. This figure combines four key visual elements: (1) electrocardiogram on admission with QTc prolongation and T-wave inversion; (2) leukonychia indicative of selenium deficiency; (3) myocardial biopsy showing sarcomere loss and vacuolation; and (4) electrocardiogram at 3-month follow-up with normalized QTc and T-waves.

## Case presentation

**Figure 1:**
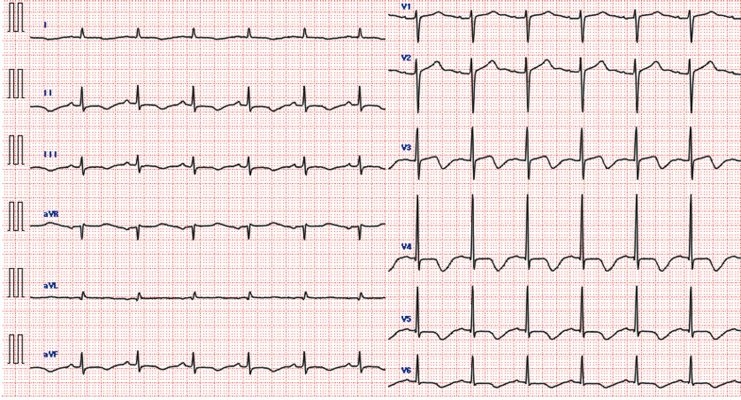
Electrocardiogram on admission. Electrocardiogram showing sinus rhythm, T-wave inversions in multiple leads, and a markedly prolonged QTc interval of 594 ms.

A 63-year-old man with a 40-year history of Crohn’s disease and multiple ileal resections was admitted with diarrhoea and loss of appetite. These symptoms contributed to progressive malnutrition and prerenal worsening in the context of chronic renal dysfunction (right renal atrophy and left hydronephrosis), ultimately leading to end-stage renal disease. His vital signs were stable, and his general physical examination findings were unremarkable. The patient had no prior history of cardiovascular disease or family history of sudden cardiac death. The patient was not taking any medications known to cause myocardial injury. Blood tests revealed elevated creatinine (7.10 mg/dL; normal range: 0.6–1.2 mg/dL) and brain natriuretic peptide (BNP) (963.4 pg/mL; normal range: <18.4 pg/mL) levels, along with hypoalbuminemia (2.1 g/dL; normal range: 4.1–5.1 g/dL), reflecting malnutrition. Electrolytes were largely within normal limits except for mildly decreased potassium (3.5 mEq/L; normal range: 3.6–4.8 mEq/L). The results of the tests for antinuclear antibodies and endocrine function were unremarkable. Electrocardiography (ECG) showed a normal sinus rhythm with a heart rate of 77 b.p.m., T-wave inversions in multiple leads, and a markedly prolonged QTc interval of 594 ms (*Figure 1*). Chest X-ray findings were within normal limits. Transthoracic echocardiography (TTE) performed 3 months earlier showed an LVEF of 52.8% and an LV end-diastolic volume (EDV) of 112 mL (see Supplementary material online, *Video S1*). However, the LVEF was diffusely reduced to 29.2% upon admission, and cardiac enlargement was noted, with the EDV increasing to 162 mL. There was no LV wall thickening or thinning, and no significant valvular abnormalities were detected (*Video 1*). Computed tomography angiography revealed no significant coronary artery stenosis, and gallium scintigraphy revealed no abnormal accumulation in the heart or other organs. Because of advanced chronic kidney disease and the associated contraindications to gadolinium-based contrast agents, cardiac magnetic resonance imaging could not be performed.

Haemodialysis was initiated. Bisoprolol (1.25 mg/day) and losartan (25 mg/day) were administered as guideline medical therapy (GDMT) for stage B heart failure according to the AHA/ACC classification. The doses were not increased due to limited blood pressure. Diuretics were not used. SGLT2 inhibitors and mineralocorticoid receptor antagonists were avoided because of end-stage renal disease and the absence of heart failure symptoms. Despite 3 weeks of dialysis and optimized medical therapy, there was no improvement in the cardiac function.

**Figure 2:**
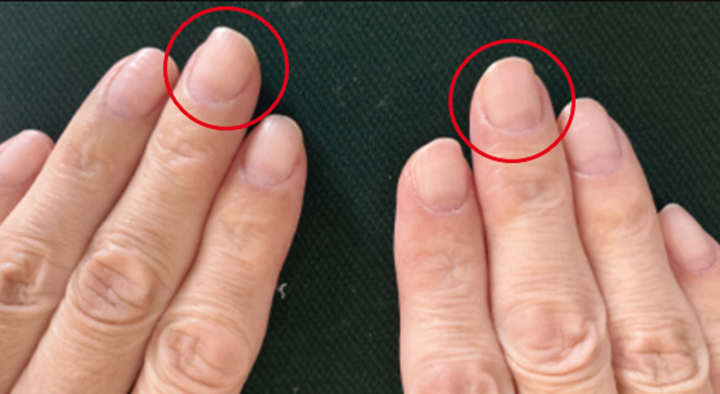
The presence of white nails. White discolouration of the nails (‘leukonychia’, circle) was detected on both hands, a characteristic finding associated with selenium deficiency.

**Figure 3:**
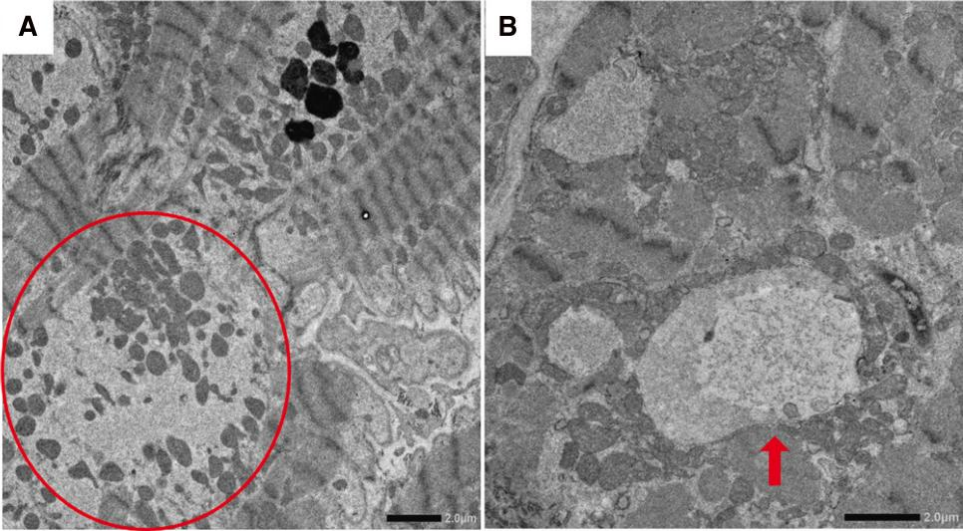
Electron microscopy of myocardial biopsy. A myocardial biopsy of the right ventricle on electron microscopy (*A* and *B*) revealed abnormal myocyte alignment, sarcomere loss (circle), and vacuole formation (arrow), consistent with selenium deficiency-induced myocardial degeneration.

Additional causes were considered (*Table 1*). Upon reviewing the patient’s history, particular attention was paid to his prior intestinal resection for Crohn’s disease and the initiation of haemodialysis, suggesting a metabolic disorder associated with impaired intestinal absorption. These are the significant risk factors for selenium deficiency. Subsequent detailed physical examination revealed leukonychia (*Figure 2*), which is a characteristic sign of selenium deficiency. Serum selenium levels were low at 5.8 µg/dL (normal range: <10.0 µg/dL). A myocardial biopsy revealed abnormal myocyte alignment, sarcomere loss, and vacuole formation on electron microscopy (*Figure 3*), consistent with selenium deficiency-induced cardiomyopathy.

**Table 1:** A summary of differential diagnoses considered in this case

Potential cause	Findings/rationale for exclusion
Hypertensive heart disease	No long-standing hypertension, no LV hypertrophy
Valvular heart disease	No significant valvular disease
Myocarditis	No signs of inflammation were observed in the myocardial biopsy
Infiltrative cardiomyopathy (e.g. amyloidosis, sarcoidosis)	No abnormal findings in the laboratory data to suggest this, and gallium scintigraphy and myocardial biopsy were also negative
Tachycardia-induced cardiomyopathy	No sustained tachyarrhythmia
Endocrine/metabolic causes (thyroid disease, adrenal disorders)	No abnormal findings in the laboratory data to suggest this
Nutritional deficiencies (thiamine, selenium)	Severe selenium deficiency (5.8 μg/dL) identified; thiamine normal
Toxic cardiomyopathy (alcohol, drugs)	No alcohol abuse or cardiotoxic drugs
Genetic cardiomyopathy	No family history of cardiomyopathy or sudden death

Comprehensive evaluation excluded valvular, infiltrative, inflammatory, and endocrine causes. Notably, profound selenium deficiency was identified as the primary aetiology.

**Figure 4:**
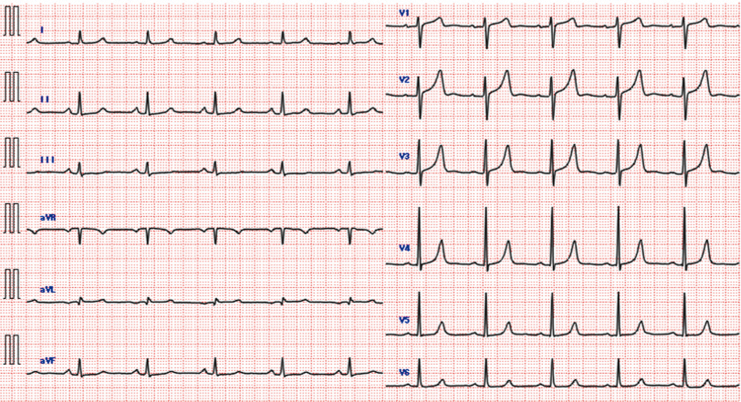
Electrocardiogram at 3-month follow-up. Electrocardiogram showing normalization of T-waves and QTc interval after selenium supplementation.

Once selenium deficiency was confirmed, supplementation was initiated with a daily intravenous infusion of 100 µg because of a concern regarding poor absorption after oral administration. After 1 week, serum selenium levels increased to 8.7 µg/dL, and BNP improved to 332.2 pg/mL. Transthoracic echocardiography showed significant recovery, with LVEF increasing from 29.2% to 43.3% and EDV decreasing to 136 mL (*Video 2*). The patient was discharged 10 days after initiating selenium supplementation and continued treatment while undergoing outpatient haemodialysis. Discharge recommendations included continuation of intravenous selenium replacement at the haemodialysis clinic with careful serum level monitoring, maintenance of low-dose bisoprolol and losartan, dietary counselling for Crohn’s disease and malnutrition, and close outpatient follow-up with repeat echocardiography and ECG assessment. Three months after discharge, LVEF further improved dramatically to 50.0%, EDV decreased to 114 mL (*Video 3*), and BNP decreased to 173.6 pg/mL. The ECG normalized (*Figure 4*), and the whitening of the nails resolved.

## Discussion

This case presented a challenging differential diagnosis of acute cardiac dysfunction. However, based on the patient’s history and physical examination results, selenium deficiency was identified as the underlying cause. Despite the extensive myocardial cell degeneration observed by electron microscopy, cardiac function markedly improved following selenium supplementation.

Non-ischaemic cardiomyopathy can have various causes. Identifying treatable aetiologies is crucial for appropriate management.^5,6^ Selenium deficiency is not explicitly listed in the current heart failure guidelines (2021 ESC and 2022 AHA/ACC/HFSA guidelines). Nevertheless, our findings support the inclusion of trace element deficiencies in the diagnostic considerations of patients with unexplained cardiomyopathy, especially when multiple risk factors are present. Selenium deficiency-induced cardiomyopathy, also referred to as Keshan disease, is a rare but potentially life-threatening condition associated with severe cardiac dysfunction if not promptly treated.^2^ Selenium deficiency may present with non-specific symptoms, often leading to delayed diagnosis.^7,8,9^ Previous studies, including the work by Frustaci *et al*., have demonstrated that selenium supplementation can lead to substantial improvement in cardiac function in patients with non-ischaemic cardiomyopathy owing to malabsorption or dialysis-related losses, supporting its potential role as a modifiable contributor to myocardial dysfunction.^10^

Selenium deficiency occurs in specific populations, such as those on long-term parenteral nutrition, dialysis, or gastrointestinal malabsorption, including those with Crohn’s disease or extensive intestinal resection.^4,10^ In patients with end-stage renal disease on haemodialysis, deficiency may be further exacerbated by restricted diets, increased selenium loss, and inflammation-induced redistribution.^2^ In our patient, the combination of Crohn’s disease, extensive ileal resection, chronic inflammation, and dialysis dependence represented a high-risk setting for profound deficiency.

In our case, QTc prolongation and T-wave inversion were noted, findings previously reported in selenium deficiency cardiomyopathy. The likely mechanism is impaired glutathione peroxidase activity with oxidative stress, causing abnormal calcium and potassium handling and delayed repolarisation. These observations underscore the need to consider trace element deficiency in unexplained repolarisation abnormalities.

Though essential for health, selenium has a narrow therapeutic window; toxicity can occur with excessive supplementation.^7,11^ The ESPEN micronutrient guideline recommends supplementation based on deficiency severity, typically 50–400 µg/day in adults.^1^ In this case, intravenous selenium at 100 µg/day led to rapid improvement in laboratory and echocardiographic parameters, confirming efficacy. Long-term supplementation with monitoring is necessary.

The pathophysiology of selenium deficiency-induced cardiomyopathy involves decreased activity of glutathione peroxidase, an antioxidant enzyme, whose impairment leads to excess reactive oxygen species (ROS) accumulation in cardiac myocytes.^3,7^ This oxidative stress triggers apoptosis and necrosis of myocytes. The heart has low catalase activity and relies predominantly on glutathione peroxidase for ROS detoxification. Histologically, selenium-deficient cardiomyopathy may resemble dilated cardiomyopathy but with distinctive features such as myofibrillolysis, cytoplasmic vacuolization, and increased apoptosis.^10^

In the present case, myocardial biopsy revealed myocyte disarray, sarcomere disruption, and vacuolization, consistent with selenium deficiency. Importantly, despite structural degeneration, cardiac function recovered significantly with supplementation, suggesting that selenium-induced myocardial injury may be partially reversible if addressed early.

In conclusion, we reported a rare case of selenium deficiency-induced cardiomyopathy characterized by marked degeneration of myocardial cells. Selenium supplementation significantly improved cardiac function despite substantial myocardial damage. These findings highlight the importance of considering selenium deficiency in patients with non-ischaemic cardiomyopathy, particularly in those with multiple risk factors such as Crohn’s disease, extensive intestinal resection, and dialysis-dependent end-stage renal disease.

## Supplementary Material

ytag184_Supplementary_Data

## Data Availability

The data underlying this article are available in the article and its online supplementary data.

## Lead author biography

Dr Tomonari Moriizumi is an eighth-year physician in the Department of Cardiology at Keio University School of Medicine in Tokyo, Japan.

## Notes

**Consent:** In accordance with COPE guidelines, written informed consent was obtained from the patient for the publication of this case report and any accompanying images or videos.

## References

[1] Berger MM, Shenkin A, Schweinlin A, Amrein K, Augsburger M, Biesalski HK, et al ESPEN micronutrient guideline. Clin Nutr 2022;41:1357–1424. 35365361 10.1016/j.clnu.2022.02.015

[2] Li Q, Liu M, Hou J, Jiang C, Li S, Wang T. The prevalence of Keshan disease in China. Int J Cardiol 2013;168:1121–1126. 23218571 10.1016/j.ijcard.2012.11.046

[3] Lubos E, Loscalzo J, Handy DE. Glutathione peroxidase-1 in health and disease: from molecular mechanisms to therapeutic opportunities. Antioxid Redox Signal 2011;15:1957–1997. 21087145 10.1089/ars.2010.3586 PMC3159114

[4] Massironi S, Viganò C, Palermo A, Pirola L, Mulinacci G, Allocca M, et al Inflammation and malnutrition in inflammatory bowel disease. Lancet Gastroenterol Hepatol 2023;8:579–590. 36933563 10.1016/S2468-1253(23)00011-0

[5] Elliott P, Andersson B, Arbustini E, Bilinska Z, Cecchi F, Charron P, et al Classification of the cardiomyopathies: a position statement from the European Society of Cardiology working group on myocardial and pericardial diseases. Eur Heart J 2008;29:270–276. 17916581 10.1093/eurheartj/ehm342

[6] Pinto YM, Elliott PM, Arbustini E, Adler Y, Anastasakis A, Böhm M, et al Proposal for a revised definition of dilated cardiomyopathy, hypokinetic non-dilated cardiomyopathy, and its implications. Eur Heart J 2016;37:1850–1858. 26792875 10.1093/eurheartj/ehv727

[7] Rayman MP. The importance of selenium to human health. Lancet 2000;356:233–241. 10963212 10.1016/S0140-6736(00)02490-9

[8] Bai S, Zhang M, Tang S, Li M, Wu R, Wan S, et al Effects and impact of selenium on human health: a review. Molecules 2024;30:50. 39795109 10.3390/molecules30010050 PMC11721941

[9] Fairweather-Tait SJ, Bao Y, Broadley MR, Collings R, Ford D, Hesketh JE, et al Selenium in human health and disease. Antioxid Redox Signal 2011;14:1337–1383. 20812787 10.1089/ars.2010.3275

[10] Frustaci A, Sabbioni E, Fortaner S, Farina M, Del Toro R, Tafani M, et al Selenium- and zinc-deficient cardiomyopathy in human intestinal malabsorption. Eur J Heart Fail 2012;14:202–210. 22186680 10.1093/eurjhf/hfr167

[11] Vinceti M, Filippini T, Cilloni S, Bargellini A, Vergoni AV, Tsatsakis A, et al Health risk assessment of environmental selenium: emerging evidence and challenges. Mol Cell Endocrinol 2017;474:31–42. 10.3892/mmr.2017.6377 PMC5428396 28339083

